# *Ixodes ricinus* abundance and its infection with the tick-borne pathogens in urban and suburban areas of Eastern Slovakia

**DOI:** 10.1186/1756-3305-6-238

**Published:** 2013-08-16

**Authors:** Lucia Pangrácová, Markéta Derdáková, Ladislav Pekárik, Ivana Hviščová, Bronislava Víchová, Michal Stanko, Helena Hlavatá, Branislav Peťko

**Affiliations:** 1 Institute of Parasitology SAS, Košice, Hlinkova 3 040 01, Slovakia; 2 Institute of Zoology SAS, Bratislava, Dúbravská cesta 9 845 06, Slovakia; 3 Slovak Hydrometeorogical Institute, Košice, Ďumbierska 26 041 17, Slovakia

**Keywords:** *Ixodes ricinus*, *Borrelia burgdorferi* sensu lato, *Anaplasma phagocytophilum*, *Neoehrlichia mikurensis*, PCR-RFLP, Lyme borreliosis, Anaplasmosis

## Abstract

**Background:**

Raising abundance of ticks and tick-borne diseases in Europe is the result of multiple factors including climate changes and human activities. Herein, we investigated the presence and seasonal activity of *Ixodes ricinus* ticks from 10 urban and suburban sites in two different geographical areas of southeastern and northeastern Slovakia during 2008–2010. Our aim was to study the abundance of ticks in correlation with the environmental factors and their infection with *Borrelia burgdorferi* sensu lato, *Anaplasma phagocytophilum* and *Neoehrlichia mikurensis*.

**Methods:**

Questing *I. ricinus* ticks were collected from ten urban and suburban sites in Eastern Slovakia. A total of 670 ticks were further analysed for the presence of *B. burgdorferi* s.l., *A. phagocytophilum* and *N. mikurensis* by molecular methods. Tick site and environmental relations were analysed using General Linear Models (LM). The differences between the number of Lyme borreliosis cases between the Košice and Bardejov regions during a ten-year period were tested by Wilcoxon matched pairs test.

**Results:**

In total, 2921 (1913 nymphs, 1008 adults) *I. ricinus* ticks were collected from 10 study sites during the main questing season. Tick activity and relative abundance differed between locations and months. Temperature and humidity were the main factors affecting the tick abundance and questing activity. Out of 670 examined ticks, 10.15% were infected with spirochetes from *B. burgdorferi* s.l. complex (represented by *B. afzelii*, *B. garinii*, *B.valaisiana* and *B. burgdorferi* s.s.), 2.69% with the *A. phagocytophilum* and 2.39% with *N. mikurensis*. The number of Lyme borreliosis cases per 100,000 inhabitants in the Bardejov region was significantly higher than in the Košice region.

**Conclusions:**

Our data indicate that the risk of infection with tick-borne pathogens in Eastern Slovakia is common since 15.2% of ticks were infected at least with one of the tested microorganisms. Even though the abundance of ticks was affected by the microclimatic conditions and the prevalence of pathogens differed between the habitats, the infection risk for humans is also affected by human activities leading to an increased contact with infected ticks.

## Background

In Europe, the changing climate and human activities in the environment have caused the changes in tick abundance together with the spread of ticks into the northern regions, urban and suburban areas as well as higher altitudes. This phenomenon is associated with the spread of tick-borne pathogens and new foci in the areas, previously free of the tick-borne diseases, which have been established [1-5]. Abiotic factors of the microclimate such as temperature, humidity, saturation or vapour pressure deficit and wind influence the survival of ticks and their questing behaviour in the habitat [6-8]. Structure of the habitat and the host availability for ticks also largely influence their phenology. The epidemiologically most important tick in Europe, *Ixodes ricinus,* transmits viral, bacterial as well as protozoan pathogens to humans and animals. The most commonly occurring and the most serious bacterial agents transmitted by this tick in Europe are spirochetes from the *Borrelia burgdorferi* sensu lato complex. They are causative agents of Lyme borreliosis, the multisystemic disorder that is maintained in natural foci in a wide spectrum of vertebrate reservoir hosts [9]. Currently 19 different genospecies belong to this complex out of which at least 9 are present in Europe [10]. The specific associations of different genospecies with the reservoir hosts as well as clinical symptomatics have been assigned [11], however, this association is not strict and differences have been observed [12]. The occurrence of Lyme borreliosis has been reported from various habitats of Europe between 35 to 60°N with the focal distribution even within small countries and thus following the occurrence of ticks [13]. The highest yearly incidence is in Central Europe, namely in Austria and Slovenia, with 130 and 136 cases per 100 000 inhabitants [13]. Another bacterial zoonotic disease, transmitted by *I. ricinus*, is granulocytic anaplasmosis caused by *Anaplasma phagocytophilum*. Anaplasmosis is the common tick-borne bacterial disease of domestic animals often causing tick-borne fever in ruminants on pastures [14]. In Europe, human cases are less common than in US [15,16]. The prevalence of *A. phagocytophilum* in questing ticks in Slovakia varies from 1.1 to 7.8% [17]. *Neoehrlichia mikurensis* is another tick-borne pathogen from the family Anaplasmataceae that attracts the attention of public health professionals in Europe. It was detected in questing ticks throughout Europe [18-21]. Rodents have been proposed as potential reservoir hosts since it was detected in blood and endothelial cells of their spleens and livers [22,23]. Recently its pathogenicity in humans was reported as it was detected in patients with septicaemia and immunosuppressed patients [24-27]. Moreover, it was detected in a chronically neutropenic dog from Germany [28].

The main aim of this study was to investigate the abundance and activity of *I. ricinus* ticks in urban and suburban areas of two cities in Eastern Slovakia in relation to the tick habitat and environmental conditions. The infection rates with the three most important tick-borne bacterial pathogens (*B. burgdoferi* s.l., *A. phagocytophilum* and *N. mikurensis*) were investigated as well. Furthermore, the occurrence of ticks and the presence of *Borrelia* were analysed in conjunction with the incidence of human cases reported to the State Health Institute during the last 10 years from both studied regions.

## Methods

### Collection of ticks

*I. ricinus* ticks were collected from ten sites (Table 1). Five model sites were selected in suburban forest and urban parks of Košice – a large urban agglomeration in southeastern Slovakia with previously known high occurrence of ticks and its infection with *Borrelia* as well as *Anaplasma*[29] and five sites were selected in Bardejov- a small town in northeastern Slovakia, with a cooler climate and very few data on presence of ticks. Ticks were collected from April till October 2008 in Bardejov area and from April till October 2010 in Košice area. Each collection was conducted using white corduroy flags for one or more hours of flagging to cover various types of land cover in each studied site. Relative abundance of ticks was calculated per one hour of flagging at each collection site and collection. After the collection, ticks were immediately immersed in tubes with 70% ethanol until the DNA was extracted. Ticks were further analysed for the presence of *B. burgdorferi* s. l., *A. phagocytophilum* and *N. mikurensis* by molecular methods.

**Table 1:** Description of tick collection sites

**Site**	**Geographical coordinates**	**Altitude**	Site group*	**Habitat type**
**Northeast- Bardejov**				
1 Smilno	49°23′05′′N 21°20′58′′E	425 m a.s.l.	C	-Birch-beech dry forest with hornbean shrubs
2 Tročany	49°11′00′′N 21°20′00′′E	345 m a.s.l.	B	-Maple-oak forest with shruby humid vegetation around pathways, close to the agricultural land
3 Raslavice	49°09′00′′N 21°19′00′′E	310 m a.s.l.	C	-Beech-oak dry forest,
4 Poštárka	49°16′15′′N 21°17′03′′E	332 m a.s.l.	B	-Suburban beech forest with shruby vegetation, in close proximity of cattle pastures
5 Bardejovské kúpele	49°19′45′′N 21°16′15′′E	283 m a.s.l.	C	-Urban beech-oak forest with park recultivation in some areas
**Southeast- Košice**				
6 Adlerova	48°74′00′′N 21°27′00′′E	321 m a.s.l.	A	- Hornbeam suburban forest with shrubby vegetation
7 Anička	48.74′00′′N, 21°25′00′′E	200 m a.s.l.	C	- Urban park with large open areas without trees
8 Botanická záhrada	48°44′49′′N 21°14′89′′E	208 m a.s.l.	B	- Urban hornbeam-oak park with shrubs
9 Verejný cintorín	48°69′00′′N 21°25′00′′E	200 m a.s.l.	B	- Urban park at the cemetery
10 Jazero	48°67′00′′N 21°31′00′′E	192 m a.s.l.	B	- Urban hornbeam forest

*Habitats were classified into three groups according to the abundance of ticks (A-highest, B-lower, C-lowest).

**Figure 1:**
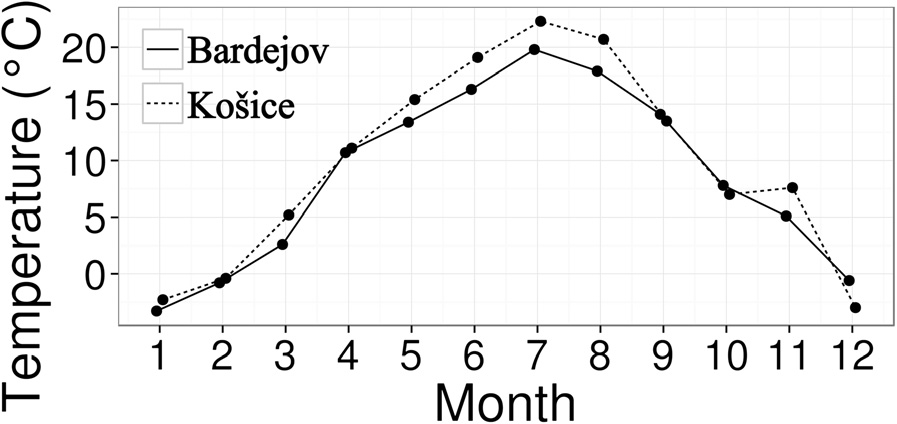
Mean monthly temperatures in Košice and Bardejov.

**Figure 2:**
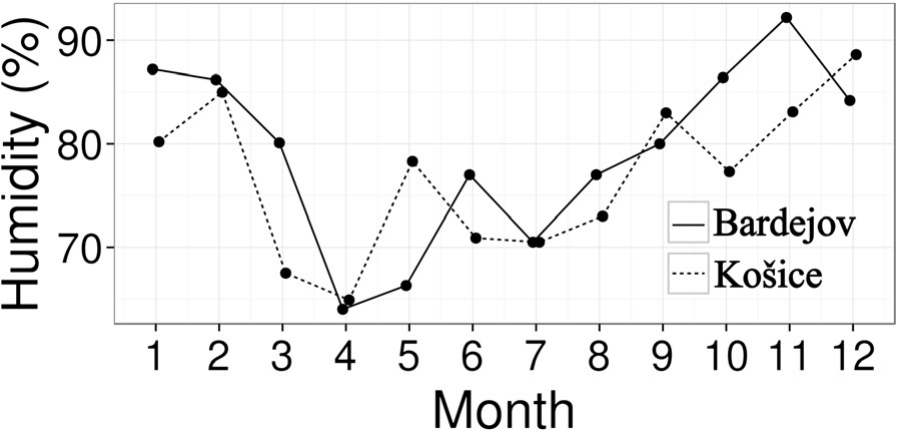
Mean monthly humidity values in Košice and Bardejov.

Saturation deficit was calculated according to Randolph *et al.*[6] and vapour pressure deficit was calculated following the approach of Li *et al*. [8]. Daily mean temperatures (Figure 1) and humidity values (Figure 2) were obtained from the Slovak Hydrometeorogical Institute in Košice from the nearest meteorogical stations (48°67′06″N; 21°23′86″E) at 229 m asl. in Košice and (49°28′47″N a 21°27′06″E) at 305 m asl., in Bardejov.

### Statistical analysis

Tick site and environmental relations were analysed using General Linear Models (LM). Basic models (with only fixed terms) were extended by random terms to deal with replications in the samples (repeated samples per sites). The response variable, tick relative abundance was (log + 1) transformed to meet the requirements of normal error distribution. To assess the influence of selected variables on tick relative abundance, a model containing fixed independent terms, humidity, temperature, saturation deficit, vapour pressure deficit, elevation and biotope type and their quadratic forms and random term site identity was constructed. To select the best model, the least significant variables were removed from the model and new models were refitted. In the case that the fitting method based on restricted maximum likelihood (REML) did not allow model comparison, models were refitted by the maximum likelihood method. These procedures were repeated until the model changed significantly and until the Akaike information criterion (AIC) was decreasing. To assess the differences between sites in tick relative abundance a model containing fixed term site and random term month was constructed. If the results of contrasts (default treatment contrast) showed similar estimates and standard errors for particular sites, these sites were pooled and the model was refitted again. The best model selection followed the steps described above. All modelling procedures followed the approach described in Zuur *et al.*[30]. The differences between the number of Lyme borreliosis cases between the Košice and Bardejov regions during a ten-year period were tested by Wilcoxon matched pairs test. Data on Lyme borreliosis case incidence in studied regions (Košice and Bardejov) were obtained from the Epidemiological Information System of Slovakia [31]. Confidence intervals (CI) for infection rates were obtained by Exact Binomial test. All statistical analyses were performed in R statistical software environment [32] and R package nlme [33].

### Molecular identification of tick-borne pathogens

Genomic DNA from each tick was isolated after its removal from ethanol and drying on filter paper by the method of alkaline-hydrolysis using 1.25% of ammonium solution, according to the previously described protocol [34]. Each tick was homogenized with a sterile pestle and negative extraction controls containing only ammonium solution were prepared for each set of DNA extraction to monitor the possible contamination. Extracted DNA from ticks was further analysed for the presence of *B. burgdorferi* s.l. complex by amplification of a 250 bp long fragment of 5S-23S (rrfA-rrlB) rDNA intergenic spacer using primers IgsA (5′CGACCTTCTTCGCCTTAAAGC′3) and IgsB (5′AGCTCTTATTCGCTGATGTA′3) [35]. For the molecular detection of *A. phagocytophilum,* PCR amplification of a 849 bp long fragment of *msp4* gene was used, with primers MAP4Ap5 (5′ATGAATTACAGAGAATTGCTTGTAGG′3) and MSP4Ap3 (5′TTAATTGAAAGCAAA TCTTGCTCCTATG′3) [36]. To detect *N. mikurensis* a 560 bp long fragment of 16S rRNA gene was amplified using IS58-594r (5′CTATCCTCTCTCGATCTCTAGT′3) and IS58-62f (5′GGAATAGCTGTTAGAAATGAC′3) primers [22]. MasterTaq DNA polymerase kit (Eppendorf AG, Hamburg, Germany) was used for PCR amplifications. A total volume of 25 μl of reaction mixture consisted of: 2.5 μl template DNA (sample), 7.6 μl of nuclease free water, 12.5 μl of PCR Master mix, and 1.2 μl of each primer (10 pmole/μl). Positive and negative controls were used in each PCR reaction. The PCR products were electrophoresed on 2% agarose gels stained with GoldView Nucleic Acid Stain (Beijing SBS Genetech, Beijing, China). Amplified fragments were visualised in a transilluminator under UV light.

In the case of *Borrelia* positive ticks, samples were further assigned to the different genospecies by RFLP method using *Tru1* restriction endonuclease (Fermentas, Vilnius, Lithuania) as described before [35].

Selected positive PCR products of the 16S rDNA fragment of *N. mikurensis* were purified by using a QIAquick PCR purification kit (Qiagen, Hilden, Germany) and sequenced in both directions with the same primers as for the PCR amplifications. Sequencing was performed at the University of Veterinary Medicine and Pharmacy in Košice; Department of Microbiology and Immunology. The complementary strands of each sequenced product were manually assembled. Sequences were compared to GenBank entries by Blast N2.2.13 [37]. The GenBank accession number for the nucleotide sequences of partial 16S rDNA of *N. mikurensis* is JN378917.

## Results

**Figure 3:**
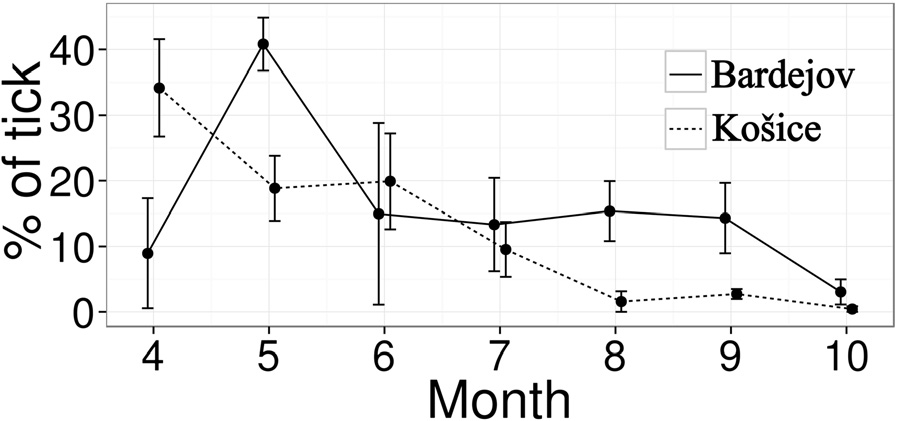
Mean tick proportion, tick proportion range during questing season at Košice (5 sites) and Bardejov (5 sites).

In total 2921 (1913 nymphs, 1008 adults) *I. ricinus* ticks were collected from 10 study sites during the main questing season of ticks. Tick activity and relative abundance per one hour of sampling differed between locations and months (Figure 3, Table 2). The highest relative abundance was observed in a suburban broadleaf forest in Košice (site no. 6) from May to July (232 – 300 ticks per hour) and the peak was recorded in June (300 ticks per hour) with the unimodal pattern. The abundance of ticks in other collection sites in Košice that were represented by urban parks (sites 7–10) was significantly lower with a unimodal pattern as well. At three sites, represented by urban parks, adult ticks were more abundant than nymphs.

**Table 2:** A relative density (RD) of ticks per one hour of collection of total sampling at model sites, a percentage proportion of developmental stage and sex

**Site**	**RD of ticks**	**% of nymphs**	**% of females**	**% of males**
**Northeast- Bardejov**				
1 Smilno	2	56	44	0
2 Tročany	15	59	21	20
3 Raslavice	24	56	22	22
4 Poštárka	12	76	14	10
5 B. kúpele	6	73	5	22
**Southeast- Košice**				
6 Adlerova	138	73	12	15
7 Anička	2	14	29	57
8 Botanická záhrada	20	77	10	13
9 Verejný cintorín	16	14	36	50
10 Jazero	15	8	41	51

In the northeastern suburban forest habitat of Bardejov (site no. 2), seasonal activity of ticks also had a unimodal pattern with the peak in May (Figure 3).

**Figure 4:**
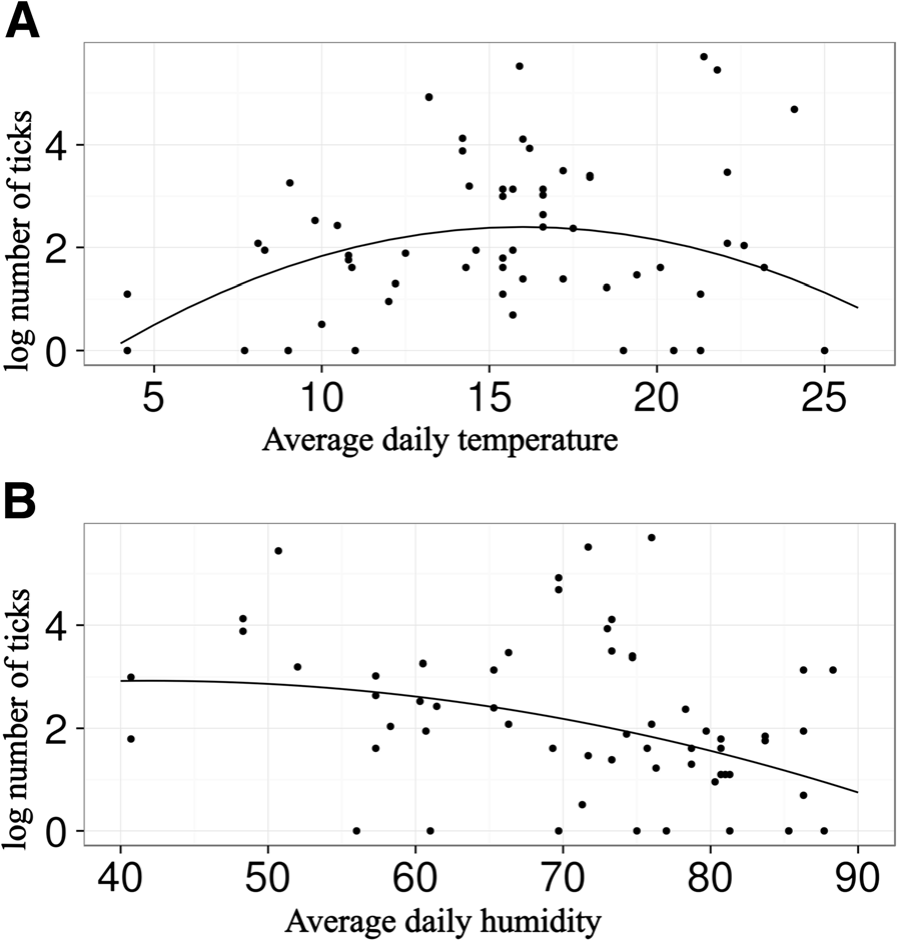
Graphical expression of the relation between average daily temperature (A), average relative daily humidity (B) and number of ticks based on the results of linear model.

The final model describing tick environmental relations consisted of two significant variables, humidity (F value 13.29, p < 0.01) and temperature (F value 1.96, p = 0.17) and temperature quadratic term (F value 11.72, p < 0.01). The tick relative abundance was higher in temperatures between 15–20°C and tick relative abundance decreased at the humidity more than 80% (Figure 4). Saturation deficit, vapour pressure deficit, site elevation and biotope type were removed from the model in the modelling procedure. Removing these variables, model AIC decreased substantially (212.19 vs. 222.44) and this step was supported by model comparison (L-ratio 7.75, p = 0.56). The model describing tick site relation showed significant differences between sites (denDF 57, F-value 38.02, p < 0.01). After pooling the sites into three groups (A, B, C) (Table 1) the model AIC decreased substantially (202.09 vs. 210.91) and this pooling was supported by model comparison (L- ratio 2.55, p = 0.92).

**Figure 5:**
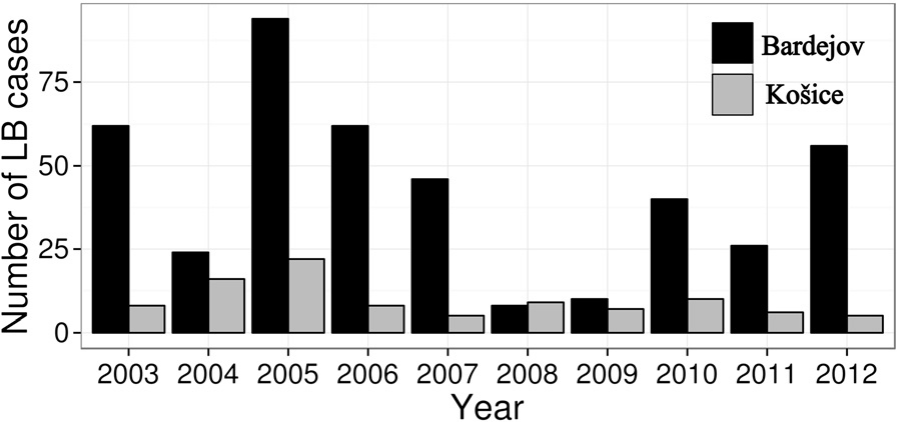
**Number of Lyme borreliosis in cases per 100 000 inhabitants in the regions Košice and Bardejov from 2003–2012 obtained from the Epidemiological Information System of Slovakia (http://www.epis.sk).**

The number of Lyme borreliosis cases per 100 000 inhabitants between the Košice and Bardejov regions were significantly different (V = 1, p < 0.01) (Figure 5), the number of cases in the latter region was higher (mean 42.8 vs. 9.60).

In total 670 ticks were tested for the presence of pathogens (*B. burgdorferi* s.l., *A. phagocytophilum* and *N. mikurensis* (Table 3). 10.15% (CI: 7.97-12.69) out of 670 examined ticks, were infected with spirochetes from *B. burgdorferi* s.l. complex. Except for one site (4), borreliae were detected at each location. RFLP analysis of positive samples revealed the presence of *B. afzelii, B. garinii, B.valaisiana, B. burgdorferi* sensu stricto and in one case mixed infection of *B. garinii* and *B. valaisiana.* The highest genetic variability of borreliae was observed in suburban forest in Košice (site 6). The mean infection rate for *A. phagocytophilum* was 2.69% (CI: 1.6-4.21) (Table 3). The occurrence of *A. phagocytophilum* was more patchy than was that for *B. burgdorferi* s.l., as it was detected only at 4 out of 10 sites. Sixteen ticks (2.39%; CI: 1.37-3.85) were infected with *N. mikurensis*. It was present at each site of five localities in the northeast (Bardejov). In contrast, in the southeast (Košice), it was detected at two out of five sites only.

**Table 3:** **Infection rate and 95% confidence interval (CI) of *N. mikurensis, A. phagocytophilum *and *B. burgdorferi *s.l. in *I. ricinus *ticks from sampling sites in Slovakia**

**Site**	**No. of ticks tested**	***N. mikurensis***	**%**	**CI**	***A. phagocytophilum***	**%**	**CI**	***B. burgdorferi *s.l.**	**%**	**CI**
**Northeast- Bardejov**	**179**	**8**	**4.47**	**(1.95-8.62)**	**3**	**1.68**	**(0.35–4.82)**	**8**	**4.47**	**(1.95–8.62)**
1 Smilno	9	1	11.11	(0.28–48.25)	0	0.00	(0.00–33.63)	3	33.33	(7.49–70.07)
2 Tročany	31	2	6.45	(0.79–21.42)	0	0.00	(0.00–11.22)	3	9.68	(2.04–25.75)
3 Raslavice	22	1	4.55	(0.12–22.84)	1	4.55	(0.12–22.84)	1	4.55	(0.12–22.84)
4 Poštárka	75	3	4.00	(0.84–11.25)	2	2.67	(0.32–9.30)	0	0.00	(0.00–4.80)
5 B. kúpele	42	1	2.38	(0.06–12.57)	0	0.00	(0.00–8.41)	1	2.38	(0.06–12.57)
**Southeast- Košice**	**491**	**8**	**1.63**	**(0.71-3.19)**	**15**	**3.05**	**(1.72–4.99)**	**60**	**12.22**	**(9.46–15.45)**
6 Adlerova	261	5	1.92	(0.62–4.41)	10	3.83	(1.85–6.93)	47	18.01	(13.5–23.22)
7 Anička	6	0	0.00	(0.00–45.93)	0	0.00	(0.00–45.93)	1	16.67	(0.42–64.12)
8 Botanical garden	79	0	0.00	(0.00–4.56)	0	0.00	(0.00–4.56)	4	5.06	(1.40–12.46)
9 Verejný cintorín	54	0	0.00	(0.00–6.60)	0	0.00	(0.00–6.60)	1	1.85	(0.04–9.89)
10 Jazero	91	3	3.30	(0.69–9.33)	5	5.49	(1.81–12.36)	7	7.69	(3.15–15.21)
**Total**	**670**	**16**	**2.39**	**(1.37–3.85)**	**18**	**2.69**	**(1.60–4.21)**	**68**	**10.15**	**(7.97–12.69)**

## Discussion

*I. ricinus* ticks are widely distributed in moderate climatic regions of Europe in both natural and urban habitats. The occurrence and recent expansion of ticks into new areas are limited by temperature and saturation deficit [5,7,38-40]. The abundance of ticks in our sites correlated to the humidity and temperature. Neither saturation deficit nor vapour pressure deficit was significant. This might be due to the differences between the microclimatic conditions at our sites and data obtained from the meteorological stations as previously observed [8]. The seasonal activity of ticks can be unimodal with one maximum peak usually in late spring or early summer or bimodal with maximum peaks in spring or summer [7,40]. We have observed unimodal patterns for all of our sites. The tick activity in northeastern sites had a maximum peak in May, one month later than for southeastern sites. This correlates with the lower temperature increase in the north as one of the significant environmental factors affecting the tick abundance observed in our models. In neighbouring Hungary, Egyed *et al.*[40] reported bimodal activity for all their sites. In the statistical model our tick sites grouped into three “site groups”– A, B, C that represented the sites with the similar tick abundance and seasonal activities. Tick group “A” had the highest abundance of ticks and only one site belonged to this group – a dense suburban forest with shrubby vegetation in southeastern Slovakia. Group “B” consisted of sites represented by urban parks in southeastern Slovakia where the vegetation was more fragmented and forested sites from the northeastern Slovakia. Third group “C” grouped together sites with the least favourable conditions for tick abundace –maintained urban park with large open spaces in southeastern Slovakia and dry suburban and urban forest in the northeast. Grouping of sites into three categories according to tick abundance showed that even small differences in the latitude (southern site vs. northern sites) with the lower daily mean temperature (Figure 1) can affect the tick abundance. Generally, less ticks were found in northeastern Slovakia in appropriate tick habitats as opposed to south.

Interestingly, the number of Lyme borreliosis cases per 100 000 inhabitants were higher for the Bardejov region in northeastern Slovakia than for Košice in southeastern Slovakia. This is probably due to larger rural areas and different outdoor human behaviour patterns in the district of Bardejov, even though Košice is the second largest city in Slovakia. The link between human activities and incidence of tick-borne diseases has already been highlighted in previous studies [41,42]. Positive correlation between the abundance of ticks and seroprevalence against borrelia and TBE was observed among farmers in neighbouring Poland [43].

Questing ticks in our study were infected with all tested zoonotic bacteria with the dominance of *B. burgdorferi* s.l. as it was detected in 10.15% (CI: 7.95-12.69) of ticks. This is in agreement with the data from Eastern Slovakia obtained by Lenčáková *et al*. [44] where 11% of ticks were *Borrelia* positive. Similar infectious rates were detected in *I. ricinus* ticks from Estonia [45]. Prevalence of *Borrelia* in neighbouring countries in Hungary [40] and Poland [43] was slightly lower. In our dataset, the highest infection rate (18%) was detected in a suburban forest in Košice, southeastern Slovakia, where the highest abundance of ticks was also recorded. Moreover, at this locality the highest diversity of *Borrelia* species was observed; probably due to a higher availability of hosts than in urban parks within the area. In the European countries, the infection rate of *A. phagocytophilum* infection in ticks is generally low. The results from the study in 11 sites in Switzerland showed 1.5% infection rate and patchy distribution [20]. We obtained similar results with 2.69% (CI: 1.6-4.2%) infection rates and it was detected at four out of ten sites. In contrast to Norway, at the areas with the higher density of the red deer, the prevalence of *A. phagocytophilum* was more consistent and higher (8.8%) [46]. *N. mikurensis*, the recently emerging pathogen, was detected in 2.23% (CI:1.37-3.85%) of ticks. Its distribution was, however, more homogenous than that for *A. phagocytophilum,* as it was detected in all northeastern sites in Bardejov. Recent studies show that *N. mikurensis* is common and frequently infects *I. ricinus* that is widely distributed in Europe [20,21].

## Conclusions

Our data indicate that the risk of infection with tick-borne pathogens in Eastern Slovakia is common since 15.2% of ticks were infected with at least with one of the tested microorganism. Even though the abundance of ticks was affected by the microclimatic conditions and the prevalence of pathogens differed between the habitats, the infection risk for humans is also affected by human activities leading to an increased contact with infected ticks.

## Competing interests

The authors declare that they have no competing interests.

## Authors’ contributions

LP and MD drafted the manuscript. LP designed all statistical models and performed the statistical analyses. LP, MD, IH, BV and MS collected ticks, isolated DNA from ticks and performed molecular detection of pathogens. MD and BP designed the study. HH collected meteorological data. All authors have read and agreed with the content of the manuscript.
